# *Bembidion* (*Ocydromus*) *terryerwini* sp. nov. from Iran (Coleoptera, Carabidae, Bembidiina)

**DOI:** 10.3897/zookeys.1044.63607

**Published:** 2021-06-16

**Authors:** Paolo Neri, Luca Toledano

**Affiliations:** 1 Via Guido Rossa 21 “San Lorenzo”, 47121, Forlì (FC), Italy Unaffiliated Verona Italy; 2 Museo Civico di Storia Naturale di Verona, Lungadige Porta Vittoria 9, 37129, Verona, Italy Museo Civico di Storia Naturale Verona Italy

**Keywords:** Central Asia, *
decorum* group, new species, *
Ocydromus
*, taxonomy

## Abstract

Bembidion (Ocydromus) terryerwini**sp. nov.** from Central Iran (Kerman and Yazd Provinces), described here, belongs to the *decorum* species group (Ocydromus Clairville, 1806). The new species is compared with the other taxa of this species group occurring in Iran and neighboring regions (Georgia, Armenia, Azerbaijan, Turkey, Iraq, Iran, South Pakistan, Afghanistan, and Turkmenistan).

## Introduction

In unidentified material of *Bembidion* Latreille, 1802 collected in Iran by our colleagues David Wrase and Peer Schnitter we discovered an unknown species of the subgenus Ocydromus Clairville, 1806 belonging to the species group of *decorum* Zenker in Panzer, 1801 due to the unicolorous elytra and the presence of microsculpture on the pronotum. This species, later found also in other collections, was compared with abundant material of almost all taxa of the group, formerly studied by Müller-Motzfeld (1986): it is described as new in this contribution and its systematic position is discussed.

## Materials and methods

The systematic treatment of the Bembidiina and the geographical acronyms follow Löbl and Löbl (2017). The body length was measured for card-mounted specimens from the front margin of the labrum to the apex of the elytra. Dissections were made using standard techniques. Genitalia and small parts were preserved in Euparal on acetate mounts fixed on the same pin as the specimens.

LT took the photographs with Nikon DSFi1 and Nikon DS-L2 on a Leica Z6 microscope, while Werner Marggi provided the photograph of *B. sokolowskyi* Fassati, 1957. The drawing of the spermatheca was made by Ivo Gudenzi.

The examined material is preserved in the following collections:

**CTVR** coll. Luca Toledano, Verona, Italy;

**DW** coll. David Wrase, Gusow Platkow (part of Zoologische Staatssammlung München, Germany);

**JM** coll. Jan Muilwijk, Bilthoven, Netherlands;

**KO** coll. Kamil Orszulik, Frýdek-Místek, Czech Republic;

**KR** coll. Karel Rébl, Nové Strašecí, Czech Republic;

**MT** coll. Marcos Toribio, Tres Cantos (Madrid), Spain;

**MHNG**Muséum d’Histoire naturelle, Genève, Switzerland;

**PN** coll. Paolo Neri, Forlì, Italy;

**PS** coll. Peer Schnitter, Halle, Germany.

## Taxonomic account

### Bembidion (Ocydromus) terryerwini sp. nov.

Taxon classificationAnimaliaColeopteraCarabidae

384D8BA9-BD0F-5160-AD5D-92C039B6407E

http://zoobank.org/E719D15B-1C66-4471-8C0E-0189212DF963

Figures 2 , 3 , 6 , 7

#### Material examined.

***Holotype***, 1 ♂, “N 29°21'14.7" E 57°20'31.7" / Iran, Prov. Kerman, Rāyen, Goroah, / Babtahoune, *Bachufer*-*Schotterbank* / 06.06.2014 2700 m / leg. Schnitter” (CTVR). We added the following label (red, printed) to the specimen: “*Bembidion (Ocydromus) terryerwini* Neri & Toledano, 2021 – Holotypus”. ***Paratypes***: 24 ♂♂ 12 ♀♀, same collecting data as the holotype (CTVR, JM, PN, PS); 4 ♂♂ 3 ♀♀, “N 29°23'35.4" E 57°25'44.4 Iran, / Prov. Kerman, Rayen, Anbaroutak, / Bachufer/Schotterbank / 06.06.2014 2350m / leg.: Schnitter” (PN, PS); 22 ♂♂ 24 ♀♀, “N 30°31'18.4" E 57°09'44.6" / Iran, Prov. Kerman, Kerman N / Darb-e-Asiab, waterfall near / Kuhpayeh, *Schlucht*-*Schotterbank* / 07.06.2014 2600 m / leg. Schnitter” (CTVR, PN, PS); 7 ♂♂ 5 ♀♀, “N 29°32'49.6" E 57°17'56.5" / Iran, Prov, Kerman, Rayen / waterfall near Kuh-e Hazar / *Bachufer*-*Schotterbank* / 05.06.2014 2920 m / leg. Schnitter” (CTVR, PS); 1 ♂ 1 ♀, “Iran, Prov. Kerman, Kuh-e / Lalehzar, 3400–3600 m / 04.06.2014, 29°27'52" N / 56°45'65" E / leg. A. Weigel” (CTVR); 1 ♂, “Iran (Kerman prov.) / Kūh-e Lālehzār vill. ca 3000–3600 m / N 29°28'41" E 56°48'21" / (river valley, river bank, / in gravel) /4.VI.2014 Wrase & Laser” (CTVR); 2 ♂♂ 3 ♀♀, “Kerman prov. 15 Km E / Korin, Kub-e-Lalehzar Mts. / (3000 m) / 18-5-2008 Iran / A. Anichtchenko leg.” (CTVR, MT); 5 ♂♂ 8 ♀♀, “Iran (Kerman Prov.) / waterfall at Darb-e-Asiab vill. / N Kerman 2600m / N 30°31'18.4"/E 57°09'44.6" / (canyon, in gravel/under stones) / 7.VI.2014 D. Wrase” (CTVR, DW, PN); 2 ♂♂ 2 ♀♀, “Iran (Kerman Prov.) / waterfall at Darb-e-Asiab vill. / N Kerman 2600m / N 30°31'18.4"/E 57°09'44.6" / (canyon, in gravel/under stones) / 2.VI.2014 B. Laser” (DW); 2 ♂♂ 2 ♀♀, “IRAN (Kerman Prov.) / waterfall at Darb-e-Asiab vill. / nr. Kuhpayeh vill. N Kerman / ca 2500 m / N 30°31'18.4"/E 57°09'44.6" / (canyon, in gravel/under stones) / 2.06.2014 D. Wrase” (DW); 3 ♂♂ 3 ♀♀, “Iran (Kerman Prov.) / Kuh-e Lalehzar / N Lalehzar vill. ca. 3000–3600 m / N 29°28'41"/E 56°48'21" / (river valley, river bank, / in gravel / 4.VI.2014 Wrase & Laser” (CTVR, DW); 1 ♂, “Iran (Kerman Prov.) / Kuh-e Hezar 2920 m / high plain above Abshar-e / Rayen water fall (SW Rayen) / N 29°32'49.6"/E57°17'56.5" / (brook bank, in gravel / sifted, from detritus) / 5.VI.2014 Wrase” (DW); 20 ♂♂ 31 ♀♀, “Iran (Kerman Prov. / Rayen distr.) Goruh Village / Babtahoune 2700 m / N 29°21'14.7"/E57°20'31.7" / (river bank, in gravel / partly with vegetation) / 6.VI.2014 Wrase & Laser” (CTVR, DW, PN); 1 ♀, “Iran (Kerman prov.) / Anbaroutak vill. nr. Rāyen / 2350 m / N 29°23'54.4" E 57°25'44.4" / (brook bank, in gravel-under / stones, partly overgrown) / 6.VI.2014 Wrase & Laser” (DW); 1 ♂, “IR Kerman / Rusk / 19-4-2008 / Muilwijk leg.” (CTVR); 22 ♂♂ 29 ♀♀, “Iran, Maimand, prov. Kerman, 160 km W Kerman, 8.5.2010 Orszulik lgt.” (CTVR, KO, KR, PN); 1 ♂ 2 ♀♀, “Iran, prov Kerman, Sarbizhan, 140 km S Kerman, 4.5.2010, Orszulik lgt.” (KO, KR); 1 ♀, “IR Kerman Darmazar Kuh-e Bahr Aseman 22.iii.2007 leg. J. Muilwijk” (JM); 1 ♂ 2 ♀♀, “IR Kerman Darmazar Kuh-e Bahr Aseman 21.iv.2008 leg. J. Muilwijk” (JM); 3 ♂♂ 1 ♀, “IR Kerman Rabor env. 22.iii.2007 leg. J. Muilwijk” (JM); 1 ♀, “IR Kerman 30 km North Baft 20.iii.2007 leg. J. Muilwijk” (JM); 1 ♀, “IR Kerman Kuh-e Hezar 21.iv.2008 leg. J. Muilwijk” (JM); 8 ♂♂ 10 ♀♀, “Iran 7.5.1999 / Sir Kūh, Yazd env. / lgt. Orszulik” (CTVR, KO, KR, PN); 3 ♂♂ 3 ♀♀, “Iran C 7.5.1999 / Yazd env. / Mt. Sir Kūh / L. Klima leg.” (CTVR, JM, KR); 1 ♂, “Iran, Yazd, Shir Kūh / 5 Km S Taft / 23.5.2008 1600–1700 m / leg. Mühle” (CTVR); 4 ♂♂ 5 ♀♀, “IR Yazd / M Sir Kou / 14-4-2008 / Muilwijk leg. 2008” (DW, JM). We added to the specimens the following label (red, printed): “*Bembidion (Ocydromus) terryerwini* Neri & Toledano, 2021 – Paratypus“.

#### Type locality.

Iran, Prov. Kerman, Rāyen, Goroah, Babtahoune 2700 m, 29°21'14.7"N, 57°20'31.7"E.

#### Comparative material.

We were able to compare the species herein described with abundant material from several collections (CTVR, PN, DW, PS, JM), except for *ispartanum nairicum* Iablokoff-Khnzorian, 1976 and *sokolowskyi* Fassati, 1957 that were compared based on the literature, by original descriptions or other publications. Of *sokolowskyi*, Werner Marggi kindly provided a photo of the holotype taken in the Coll. Fassati. We also examined the following type material: 1 ♂, paratype of *B. semilotum* Netolitzky, 1911 “Sultanabad / Th. Strauss [printed] // *semilotum* [handwritten] / det. Netolitzky [printed] // CO / TYPUS [red, printed]” (CTVR), 2 ♀♀. paratypes of *B. hiekei* Müller-Motzfeld, 1986 “Turkm., Kopet Dag / Salzquelle 2 Km v. / Tschuli b.Firjusa / 18.9.76, leg. F. Hieke [printed] // PARATYPUS [red, handwritten] // *Bembidion* [printed] / *hiekei* n.sp. [handwritten] // det. Müller 1985 [printed]” (PN).

#### Diagnosis.

A Bembidion of the subgenus Ocydromus and member of the *decorum* group; very similar to *B. hiekei* Müller-Motzfeld, 1986, but recognizable from the latter by a less transverse pronotum with narrow lateral gutter and punctured anterior transverse impression and adjacent area.

**Figures 1–7.**
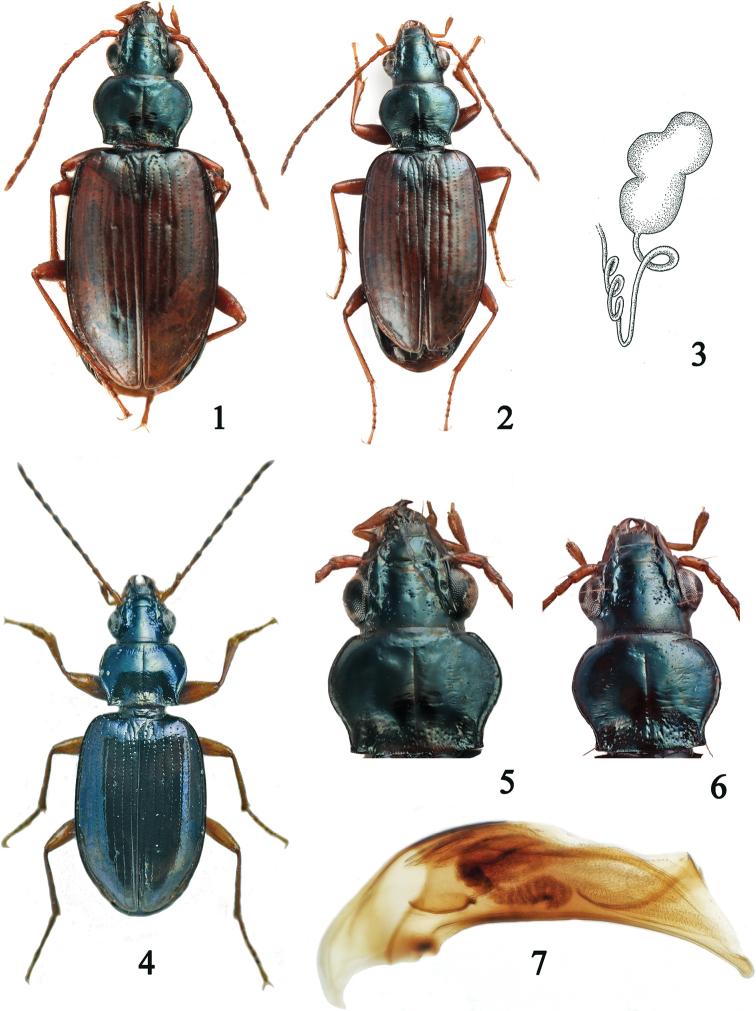
**1** habitus of Bembidion (Ocydromus) hiekei Müller-Motzfeld, paratype, (PN), 5.90 mm **2** habitus of B. (O.) terryerwini sp. nov., holotype (CTVR), 5.20 mm **3** spermatheca of B. (O.) terryerwini sp. nov., paratype (PN) **4** habitus of B. (O.) sokolowskyi Fassati, holotype, (MHNG), 5 mm **5** pronotum of Bembidion (Ocydromus) hiekei Müller-Motzfeld, paratype, (PN) **6** pronotum of B. (O.) terryerwini Neri & Toledano, sp. nov., holotype (CTVR) **7** median lobe of the aedeagus of B. (O.) terryerwini sp. nov., holotype (CTVR), 1.02 mm.

#### Description of holotype

**(Fig. 2).** Total body length 5.20 mm. ***Coloration***: head and pronotum black, with green metallic reflections, elytra brown, scutellar area blackish brown and sutural interval brown at the internal side. Antennae yellow-orange, only slightly darkened from the apical half of antennomere 4. Palps yellow-orange. Legs yellow-orange with femora with faint brown reflections.

***Head***: maximum width, including eyes, 1.07 mm; interocular distance 0.62 mm; smooth, punctured in the median portion of the frons at the posterior half of the eye; microsculpture present in the posterior portion of the neck. Eyes markedly protruding, temples very short, almost absent. Wide frontal furrows, with a few punctures. Antennae long 2.68 mm.

***Pronotum*** (Fig. 6): length along the mid line 1.03 mm; width of the anterior margin 0.87 mm, maximum width 1.26 mm, width of base 0.93 mm; pronotal width/pronotal length ratio 1.22; slightly transverse; posterior margin slightly convex at middle, oblique towards the hind angles; anterior margin rectilinear with angles evidently bent ventrally; sides entirely rebordered, narrowing evidently and markedly sinuate towards base with which they form a large right angle, despite of the oblique sides of the base; lateral gutter narrow, of homogeneous width; basal foveae subquadrate, with scattered punctures and long laterobasal carina; median line sharp; anterior transverse impression and neighboring area with numerous (about 20) very sharp punctures; basal transverse impression rugose-punctate. Microsculpture in short, polygonal transverse sculpticells, barely visible on disc.

***Elytra***: length 3.20 mm, maximum overall width, slightly beyond the middle, 2.00 mm; overall shape slightly ovoid with evident shoulders; completely microsculptured with sharp transverse polygonal sculpticells; intervals flat; striae and punctation evident but superficial, visible also near the apex but disappearing at apex. Macropterous species.

#### Male genitalia.

Medium-small sized (1.02 mm), with ventral margin only slightly concave and apical fourth bent ventrally; endophallus almost completely included in the median lobe; extremely elongated apex.

#### Intraspecific variability.

The paratypes generally match with the holotype in color and morphology. The brown color of the elytra may be more or less intense, from reddish to dark brown; the palps may be slightly darkened at apex. The pronotum may be slightly more transverse. The microsculpture of the pronotal disc may be more or less visible; the fine punctation at the anterior transverse pronotal impression, always present and sharp, may show a variable number of punctures. The ♂♂ are long from 4.70 to 5.70 mm and the ♀♀ from 4.80 to 5.70 mm. The length of aedeagus (Fig. 7) ranges from 1.00 to 1.13 mm, the reservoir of the spermatheca (Fig. 3) is 0.23 mm long.

#### Derivatio nominis.

This beautiful species is named in memory of the late Terry Erwin (1940–2020), excellent, very kind, collaborative, and friendly man, and excellent globally acknowledged entomologist, who contributed to the taxonomic research of the tribe Bembidiini through numerous publications (e.g., Erwin 1971, 1972, 1973, 1974a, b, 1975, 1976, 1977, 1978, 1982, 1984; Erwin and Kavanaugh 1980, 1981, 1999: Erwin et al. 2010: Kavanaugh and Erwin 1992).

#### Distribution.

The new species is known from the Kerman and Yazd Provinces, in central Iran.

#### Comparative notes.

The species of *decorum* group currently reported from Iran and neighboring regions are eleven. The *decorum* group includes species with pronotum microsculptured at least at sides and elytra unicolorous greenish, bluish metallic, or brown. Eleven species of this group are currently known from Iran and neighboring regions: Bembidion (Ocydromus) decorum bodemeyeri K. Daniel & J. Daniel, 1902 (Bulgaria, Greece, Macedonia, Turkey); B. (O.) decorum subconvexum K. Daniel & J. Daniel, 1902 (Azerbaijan, Armenia, Georgia, Russia: South European Territory, Iran, Turkey); B. (O.) siculum smyrnense Apfelbeck, 1904 (Albania, Bosnia Herzegovina, Bulgaria, Croatia, Greece, Macedonia, Romania, Turkey, Ukraine, Yugoslavia A: Cyprus, Israel, Lebanon, Syria, Turkey); B. (O.) siculum durudense Marggi & Huber, 1999 (Azerbaijan, Armenia, Georgia, Russia: South European Territory, Iran, Turkey); B. (O.) ispartanum ispartanum Netolitzky, 1930 (Armenia, Iran, Israel, Turkey); B. (O.) ispartanum nairicum Iablokoff-Khnzorian, 1976 (Armenia, Georgia); B. (O.) zolotarewi Reitter, 1910 (Armenia, Georgia, Russia: South European Territory, Iran); B. (O.) semilotum Netolitzky, 1911 (Armenia, Iran, Turkey); B. (O.) hiekei Müller-Motzfeld, 1986 (Iran, Turkmenistan); B. (O.) sokolowskyi Fassati, 1957 (Afghanistan); B. (O.) hasurada pagmanicum Fassati, 1959 (Afghanistan). Their aedeagi, except for *sokolowskyi*, are well documented by Müller-Motzfeld (1986). *Bembidion terryerwini* is distinguishable from the above-mentioned species, excluding *hiekei*, by the brown elytral color; from *hiekei* (Figs 1 and 5) by the less transverse pronotum with narrow lateral gutter and the anterior transverse impression and neighboring area with several punctures; from *hasuradapagmanicum* by the elytral striae less deeply impressed; from *semilotum*, *ispartanumispartanum*, and *siculumdurudense* by the yellow-orange femora with faint brown reflections and by the antennae slightly darkened from the fourth antennomere; from *ispartanumnairicum* by the antennae with the first three antennomeres light; from *siculumsmyrnense* and *siculumdurudense* by the structure of the endophallus and the not darkened femora; from *zolotarewi* by the elytral margins not evidently rounded; from *decorumbodemeyeri* and *decorumsubconvexum* by the elytra less deeply punctate-sulcate and by the structure of the endophallus; from *sokolowskyi* (Fig. 4) by the less transverse pronotum with base slightly oblique towards the angles and by the femora not darkened.

## Supplementary Material

XML Treatment for Bembidion (Ocydromus) terryerwini
